# Auditory stimulation of opera music induced prolongation of murine cardiac allograft survival and maintained generation of regulatory CD4^+^CD25^+ ^cells

**DOI:** 10.1186/1749-8090-7-26

**Published:** 2012-03-23

**Authors:** Masateru Uchiyama, Xiangyuan Jin, Qi Zhang, Toshihito Hirai, Atsushi Amano, Hisashi Bashuda, Masanori Niimi

**Affiliations:** 1 Department of Cardiovascular Surgery, Juntendo University Hospital, 2-1-1 Hongo, Bunkyo-ku, Tokyo 113-8421, Japan; 2 Department of Surgery, Teikyo University, 2-11-1 Kaga, Itabashi-ku, Tokyo 173-8605, Japan; 3 Department of Immunology, Juntendo University Hospital, 2-1-1 Hongo, Bunkyo-ku, Tokyo 113-8421, Japan; 4 Department of Cardiovascular and Thoracic Surgery, The 4th Affiliated Hospital of Harbin Medical University, 37 Yiyuan Street, Nangang District, Harbin, Heilongjiang Province, China; 5 Department of Urology, Tokyo Women's Medical University, 8-1 Kawata-cho, Shinjuku-ku, Tokyo 162-8666, Japan

**Keywords:** Opera music, Regulatory cells, Cardiac transplantation, Mouse

## Abstract

**Background:**

Interactions between the immune response and brain functions such as olfactory, auditory, and visual sensations are likely. This study investigated the effect of sounds on alloimmune responses in a murine model of cardiac allograft transplantation.

**Methods:**

Naïve CBA mice (H2^k^) underwent transplantation of a C57BL/6 (B6, H2^b^) heart and were exposed to one of three types of music--opera (*La Traviata*), classical (Mozart), and New Age (Enya)--or one of six different single sound frequencies, for 7 days. Additionally, we prepared two groups of CBA recipients with tympanic membrane perforation exposed to opera for 7 days and CBA recipients exposed to opera for 7 days before transplantation (pre-treatment). An adoptive transfer study was performed to determine whether regulatory cells were generated in allograft recipients. Immunohistochemical, cell-proliferation, cytokine, and flow cytometry assessments were also performed.

**Results:**

CBA recipients of a B6 cardiac graft that were exposed to opera music and Mozart had significantly prolonged allograft survival (median survival times [MSTs], 26.5 and 20 days, respectively), whereas those exposed to a single sound frequency (100, 500, 1000, 5000, 10,000, or 20,000 Hz) or Enya did not (MSTs, 7.5, 8, 9, 8, 7.5, 8.5 and 11 days, respectively). Untreated, CBA mice with tympanic membrane perforations and CBA recipients exposed to opera for 7 days before transplantation (pre-treatment) rejected B6 cardiac grafts acutely (MSTs, 7, 8 and 8 days, respectively). Adoptive transfer of whole splenocytes, CD4^+ ^cells, or CD4^+^CD25^+ ^cells from opera-exposed primary allograft recipients resulted in significantly prolonged allograft survival in naive secondary recipients (MSTs, 36, 68, and > 100 days, respectively). Proliferation of splenocytes, interleukin (IL)-2 and interferon (IFN)-γ production was suppressed in opera-exposed mice, and production of IL-4 and IL-10 from opera-exposed transplant recipients increased compared to that from splenocytes of untreated recipients. Flow cytometry studies showed an increased CD4^+^CD25^+ ^Forkhead box P3 (Foxp3)^+ ^cell population in splenocytes from those mice.

**Conclusion:**

Our findings indicate that exposure to opera music, such as La traviata, could affect such aspects of the peripheral immune response as generation of regulatory CD4^+^CD25^+ ^cells and up-regulation of anti-inflammatory cytokines, resulting in prolonged allograft survival.

## Background

Music has an important role in all human cultures and has been found to have direct and indirect effects on physiologic functions and clinical symptoms. Music can improve performance of reasoning tasks [1], reduce stress, enhance feelings of comfort and relaxation, provide a distraction from pain, and improve the results of clinical therapy [2]. Since World War II, the use of music therapy, which is defined as prescribed exposure to music to aid in preventing or ameliorating physical [3] and psychological problems [4], has become established internationally in a variety of health care fields. These include psychiatry [5], drug and alcohol rehabilitation [6], developmental disability therapy [7], geriatrics [8], palliative care [9], general surgery [10], and oncology [11].

Considerable research on music therapy, especially as used in acute care settings, has been performed. For example, among a group of patients recovering from a myocardial infarction, those who received music therapy had anxiety scores that were significantly lower than those of patients given routine care [12]. Ezzone et al. [3] reported that music was an effective adjunct to administration of pharmacologic antiemetic agents for lessening nausea and vomiting. Sahler et al. [4] investigated that music therapy mitigated pain and nausea, the two most common side effects of transplantation, in patients undergoing bone marrow transplantation. More recently, music therapy has been found to help in reducing agitation in patients with Alzheimer's disease [13]. Chuang et al. [11] reported preliminary evidence suggesting that music therapy may promote relaxation and increase parasympathetic nervous system activity in cancer survivors. In the transplantation field, however, the use of music therapy remains controversial, and little is known about mechanisms by which such treatment might modulate alloimmune responses. The current study investigated the effect of sounds on alloimmune responses in a murine model of cardiac allograft transplantation.

## Methods

### Animals

Male C57BL/6 (H2^b ^[B6]), CBA (H2^k^), and BALB/c (H2^d^) mice that were 8 to 12 weeks of age were purchased from Sankyo Ltd (Tokyo, Japan), housed in conventional facilities at the Biomedical Services Unit of Teikyo University, and used in accordance with the guidelines for animal experimentation approved by the Animal Use and Care Committee of the university and the "Principles of laboratory animal care" (NIH publication, vol 25, no. 28, revised 1996).

### Heart Transplantation and Tympanic membrane perforation

Heart transplantation was conducted as described previously [14,15]. Postoperatively, cardiac graft function was assessed daily by palpating the heart for evidence of contraction. Rejection was defined as complete cessation of the heartbeat and confirmed by direct visualization and histologic examination of the graft. Tympanic membrane perforation was conducted by means of tweezers, and the loss of tympanic membrane was confirmed by visual observation.

### Exposure to Music

CBA recipients of cardiac allografts were randomly assigned to one of five groups, which were either not exposed to music (no-treatment group) or were exposed to opera (*La Traviata *by Giuseppe Verdi, Royal Opera House Covent Garden Chorus and Orchestra, conducted by Sir Georg Solti, 448 119-2; Decca, London, United Kingdom [UK], 1995); classical music (*The Ultimate All Mozart*, Berlin Philharmonic, POCG-30044; Polydor, Tokyo, Japan, 1999); New Age music (*Paint the Sky with Stars: The Best of Enya*, Enya, WPCR-2345; Warner Music Japan, Tokyo, 1997); or one of six different sound frequencies (100, 500, 1000, 5000, 10,000, or 20,000 Hz) from the day of transplantation until 6 days afterward. Some of the frequencies were obtained by using computer software kindly provided by Tetsuji Katsuda (Chief Director, Asahi Music Laboratory, Tokyo; http://www.asahi-net.or.jp/~HB9T-KTD/music/Japan/Soft/SpeakerCheck.html). In addition to above five groups, we prepared two groups of CBA recipients with tympanic membrane perforation exposed to opera (*La Traviata*) from the day of transplantation until 6 days afterward and CBA recipients exposed to opera for 7 days before transplantation (pre-treatment group).

All experiments were conducted in an environment in which a cycle of 12 h of light and 12 h of darkness and a room temperature of 24°C were maintained and the level of ambient noise (such as that produced by the air conditioner) was about 40 dB (the sound level in the no-treatment group). The source of the sound in mice exposed to either the sound frequencies or music was about 1 m from the cages housing the animals, and the sound level was about 60 dB. In each of the three music-exposed groups, the musical selection was played repeatedly on a CD player with an amplifier for 24 h/day.

### Immunohistochemical Studies of Harvested Grafts

Cardiac grafts transplanted into untreated mice and mice exposed to opera music were removed 14 days after transplantation respectively and studied immunohistochemically with use of double immunostaining. Fresh 4-μm-thick graft cryosections were incubated with anti-Forkhead box P3 (Foxp3) (kindly provided by Professor Kenjiro Matsuno [16], Dokkyo Medical University, Tochigi, Japan) polyclonal antibody; incubated with alkaline phosphatase (ALP)-conjugated anti-rabbit Ig (712-055-152; Jackson ImmunoResearch Laboratories) for anti-Foxp3; and developed blue with Vector Blue (Vector Laboratories, Burlingame, CA). Cryosections were then incubated with rabbit anti-mouse type IV collagen polyclonal antibody (LB1403; Cosmo Bio, Tokyo) and peroxidase-conjugated anti-rabbit Ig (55693; Mitsubishi Chemical, Tokyo) and then developed brown with diaminobenzidine (Vector Laboratories).

### Adoptive Transfer Studies

Adoptive transfer studies were conducted to determine whether regulatory cells were generated in mice exposed to opera music. Thus, 10 days after transplantation of B6 hearts into primary CBA recipients exposed to opera music for 7 days after grafting, splenocytes (5.0 × 10^7^) from primary recipients with functioning allografts were adoptively transferred into naïve secondary CBA recipients by means of intravenous injection into the penile vein. Immediately afterward, the secondary recipients underwent transplantation of a B6 (donor-specific) or BALB/c (third-party) heart. In some experiments, CD4^+ ^cells were purified from the spleens of primary transplant recipients by positive selection using a magnetically activated cell sorter (MACS) and CD4 microbeads (Miltenyi Biotec, Auburn, CA; purity > 98%), and CD4^+ ^cells (2.0 × 10^7^) were adoptively transferred into naïve secondary recipients, which then immediately underwent transplantation of a B6 heart. In other experiments, CD4^+^CD25^+ ^cells were purified from the spleens of primary recipients exposed to opera by using a MACS and a mouse CD4^+^CD25^+ ^regulatory T-cell isolation kit (Miltenyi Biotec). CD4^+^CD25^+ ^cells (2.0 × 10^6^) were then adoptively transferred into naïve secondary recipients, which then immediately underwent transplantation of a B6 heart.

### Mixed Leukocyte Cultures

In other mixed lymphocyte culture (MLC) studies [17], the responder cells were splenocytes from naïve CBA mice or from untreated or opera-exposed CBA mice that had undergone transplantation of a B6 heart 14 days earlier. The stimulator cells were B6 (allogeneic) or CBA (syngeneic) splenocytes treated with 100 μg/ml mitomycin C (MMC; Kyowa Hakko, Osaka, Japan) for 30 min at 37°C. The responder cells (2.5 × 10^6^/ml) were co-cultured with the stimulator cells (5 × 10^6^/ml) in complete medium in a humidified 5% CO_2 _atmosphere (CH-16M; Hitachi, Tokyo) at 37°C in 96-well, flat-bottomed tissue-culture plates (Iwaki Scitech Division, Tokyo) for 4 days. Maximum proliferation of naïve CBA splenocytes (responder cells) against B6 splenocytes (stimulator cells) treated with MMC occurred on the fourth day of MLCs. Proliferation was assessed by using an ELISA for BrdU incorporation (Biotrak, version 2; Amersham, Little Chalfont, UK) according to the manufacturer's instructions [18].

### Cytokine Assays

ELISAs were also performed to assess levels of interleukin (IL)-2, IL-4, IL-10, and interferon (IFN)-γ in the supernatant of the MLCs on day 4. The capture monoclonal antibody (mAb) (JES5-2A5), detection mAb (JES5-16E3), and recombinant standard for IL-10 were from BD Biosciences. The capture and detection mAbs for IL-2 (JES6-1A12 and JES6-5H4, respectively), IL-4 (BVD-1D11 and BVD-24G2), and IFN-γ (R4-6A2 and XMG1.2) were from Caltag Laboratories (Burlingame, CA). Recombinant standards for IL-2, IL-4, and IFN-γ were from PeproTech (London, UK).

### Flow Cytometry Analysis of CD4, CD25, and Foxp3 expression

Splenocytes were obtained from naïve CBA mice and from opera-exposed and untreated cardiac allograft transplant recipients 1, 2, and 4 weeks after transplantation. The cells were stained with fluorochrome-conjugated anti-CD4 or anti-CD25 mAb (RM4-5 and PC61, respectively; BD Biosciences) or anti-mouse Foxp3 mAb (FJK-16s; eBioscience, San Diego, CA), as well as their isotype controls (eBioscience). The stained cells were analyzed by using a FACS Canto2 system (BD Biosciences). The number of CD4^+^CD25^+^Foxp3^+ ^cells and the percentage of CD4^+^CD25^+^Foxp3^+ ^in CD4^+ ^cells was determined.

### Statistical Analysis

Cardiac allograft survival in two experimental groups was compared by using log rank test. In the cell-proliferation, cytokine, and flow cytometry studies, the difference between two groups was assessed by using unpaired Student *t *tests. A *P*-value < 0.05 was considered significant.

## Results

### Survival of Fully Mismatched Cardiac Allografts in Mice Exposed to Opera Music

**Figure 1:**
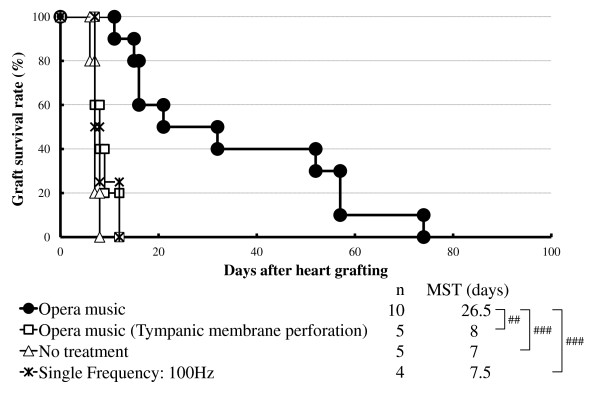
**Exposure to opera music results in the prolongation of cardiac graft survival**. Cardiac graft survival in CBA recipients of a B6 heart that were exposed to opera music or a single sound frequency (100 Hz) from the day of transplantation to 6 days afterward, untreated allograft recipients, or allograft recipients with tympanic membrane perforation exposed to opera from the day of transplantation to 6 days afterward. MST, median survival time. ##*P *< 0.005 and ###*P *< 0.001 for difference between two groups.

CBA recipients of B6 cardiac allografts that were given either no treatment, pre-treatment or were exposed to one of six single sound frequencies rejected their grafts acutely (Table 1), as did CBA mice with tympanic membrane perforation exposed to opera (median survival time [MST], 8 days; Figure 1). In contrast, CBA allograft recipients exposed to opera or classical music from the day of transplantation until 6 days afterward had significantly prolonged survival of their B6 grafts (MSTs, 26.5 and 20 days, respectively; *P *< 0.001 and *P *< 0.005 for difference between either music group compared with the no-treatment group; Figure 1 and Table 1). Mice exposed to New Age music had little prolongation of allograft survival (MST, 11 days; *P *< 0.05 compared with the no-treatment group; Table 1). These results indicate that exposure to opera music may induce more hyporesponsiveness to cardiac allografts than other two music.

**Table 1:** Cardiac allograft survival in mice exposed to music or single sound frequency*^a^*

Group	N	Individual STs (d)	MST (d)
No exposure (no treatment)	5	6, 7, 7, 7, 8	7
Opera (pre-treatment)	4	7, 8, 8, 11	8
Mozart	5	19, 20, 20, 83, 84	20
Enya	5	7, 10, 11, 11, 19	11
Frequency			
100 Hz	4	7, 7, 8, 12	7.5
500 Hz	4	7, 8, 8, 10	8
1000 Hz	4	7, 8, 10, 10	9
5000 Hz	4	7, 8, 8, 11	8
10,000 Hz	4	7, 7, 8, 18	7.5
20,000 Hz	4	7, 8, 9, 17	8.5

*^a ^*All exposures were at 60 dB; there were no significant differences between the no-exposure group and any of the single-frequency groups on log rank test.

STs, allograft survival times; MST, median allograft survival time.

### Generation of Regulatory Cells in Mice Exposed to Opera Music

**Figure 2:**
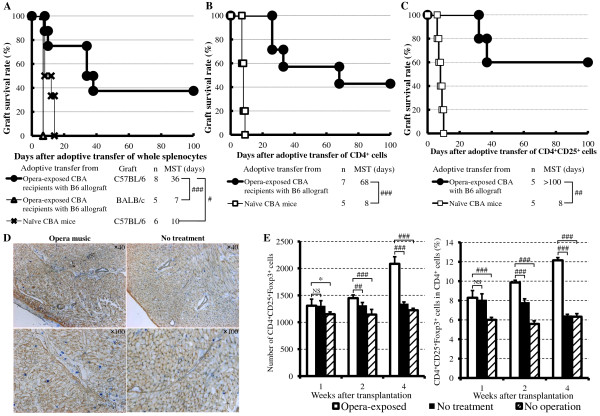
**Evidence of generation of regulatory cells in CBA allograft recipients exposed to opera music**. **A,B,C**, Cardiac allograft survival after adoptive transfer of whole splenocytes (A), CD4^+ ^cells (B), or CD4^+^CD25^+ ^cells (C). MST, median survival time. *#P *< 0.01, *##P *< 0.005 and *###P *< 0.001 for difference between two groups. **D**, Forkhead box P3 (Foxp3)^+ ^cells with use of double immunostaining. In Figure 2D, the left-hand panels show samples obtained from mice exposed to opera music (magnification × 40 and × 100) and the right-hand panels show samples from untreated mice (magnification × 40 and × 100). **E**, CD4, CD25, and Foxp3 expression in splenocytes as determined by flow cytometry 1, 2, and 4 weeks after transplantation. *P > 0.05, ##*P *> 0.005 and ###*P *< 0.001 for difference between two groups. NS, not significant.

We previously found that some anti-inflammatory or immuno-modulatory agents induce hyporesponsiveness to fully allogeneic grafts by means of generation of regulatory cells [19-21]. In the current study, naïve secondary CBA allograft recipients given adoptive transfer of splenocytes, CD4^+ ^cells or CD4^+^CD25^+ ^cells from opera-exposed primary CBA recipients 10 days after heart transplantation had significantly prolonged survival of B6 hearts (MSTs, 36, 68, and > 100 days, respectively; *P *< 0.01, *P *< 0.001, and *P *< 0.005; Figure 2A, B and 2C). In contrast, naïve secondary CBA recipients given adoptive transfer of splenocytes, CD4^+ ^cells, or CD4^+^CD25^+ ^cells from naïve CBA mice rejected B6 hearts acutely (MSTs, 10, 8, and 8 days, respectively). Moreover, when whole splenocytes from opera-exposed primary CBA transplant recipients with functioning B6 allografts were adoptively transferred into naïve secondary CBA recipients that then immediately underwent transplantation of a BALB/c heart, the BALB/c allografts were rejected acutely (MST, 7 days; Figure 2A). These data indicate that exposure to opera generated regulatory cells in the primary allograft recipients which may have been donor specific and that one of the regulatory populations consisted of CD4^+^CD25^+ ^cells.

The immunohistochemical studies showed that cardiac allografts from opera-exposed recipients had more Foxp3^+ ^cells than those from untreated mice (Figure 2D). Flow cytometry studies showed that the population of CD4^+^CD25^+^Foxp3^+ ^cells was increased in the spleens of opera-exposed recipients compared with those of naïve CBA mice (Figure 2E). These data suggest that the CD4^+ ^regulatory cells contained a population that was CD4^+^CD25^+^Foxp3^+^.

### Cell Proliferation and Cytokine Production in Mice Exposed to Opera Music

**Figure 3:**
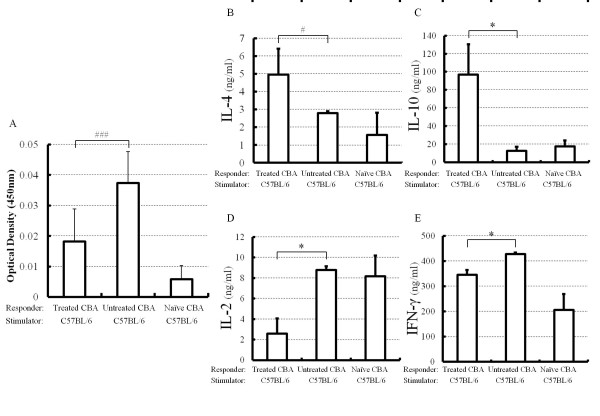
**Evidence of induction of alloproliferative hyporesponsiveness by opera music**. **A**, Results of cell-proliferation assays in mixed leukocyte cultures (MLCs). The data shown are mean ± SD values. ###*P *> 0.001 for difference between two groups. **B,C,D,E**, MLC studies of cytokines. *P > 0.05 and #*P *> 0.01 for difference between two groups.

Proliferation of splenocytes from CBA transplant recipients exposed to opera was markedly suppressed compared with that of splenocytes from untreated recipients or naive CBA mice (Figure 3A). Levels of IL-4 (Figure 3B) and IL-10 (Figure 3C) in splenocytes from CBA mice exposed to opera were significantly higher than those in splenocytes from untreated or naïve syngeneic mice (*P *< 0.01 and *P *< 0.05, respectively). On the other hand, levels of IL-2 (Figure 3D) and IFN-γ (Figure 3E) were considerably decreased in opera-exposed recipients compared with untreated recipients (*P *< 0.05).

## Discussion

In the current study, one week of exposure to opera and Mozart music apparently induced much more significantly prolonged survival of fully MHC-mismatched cardiac allografts in a murine model compared with New Age music Enya. In contrast, CBA recipients exposed to one of six single sound frequencies or pre-treatment rejected acutely. According to above data, opera music itself may not induce Treg and also music genre may be unrelated to the graft results. However, CBA pre-treated with tympanic membrane perforation rejected acutely, suggesting that auditory brain function may play an important role of Treg induction and graft prolongation.

There are several possible mechanisms by which exposure to opera might have induced increased allograft survival in our model. One is that the exposure resulted in generation of regulatory cells. Acquisition of hyporesponsiveness to an allograft is a dynamic, multistep process involving many mechanisms, including immune regulation, deletion, anergy, and ignorance [22]. Among these, immune regulation, the control of alloimmune responses by regulatory cells, is considered as one of the most important. Active suppression by regulatory cells is involved in the induction and maintenance of self-tolerance [23] and unresponsiveness to allografts [24]. In our adoptive transfer studies, most naïve secondary CBA transplant recipients given splenocytes from opera-exposed primary CBA recipients with functioning B6 cardiac allografts had significantly prolonged survival of their allografts (MST, 36 days). Furthermore, adoptive transfer of CD4^+ ^or CD4^+^CD25^+ ^cells from opera-exposed primary transplant recipients resulted in longer or indefinite prolongation of allograft survival in secondary recipients (MSTs, 68 and > 100 days, respectively). These data suggest that exposure to opera generated regulatory cells in the primary recipients and that the regulatory population contained CD4^+^CD25^+ ^cells. In addition, flow cytometry studies showed that the number of CD4^+^CD25^+^Foxp3^+ ^cells and the percentage of CD4^+^CD25^+^Foxp3^+ ^cells in CD4^+ ^cells were increased in the primary allograft recipients.

Moreover, our MLC finding of up-regulation of IL-10 production by splenocytes in opera-exposed allograft recipients suggests that IL-10 contributed to the generation of regulatory cells. IL-10 has anti-inflammatory and suppressive effects on most hematopoietic cells, and it plays a crucial role not only in the function of regulatory cells but also in their generation [25]. We previously demonstrated the importance of IL-10 in generating regulatory cells in our murine cardiac transplantation model [26]. Thus, it is probable that in the current study, it was through the up-regulation of IL-10 that exposure to opera resulted in induction of CD4^+^CD25^+ ^regulatory cells. Also, an anti-inflammatory effect may be induced through regulatory cells. Our histologic studies of allografts obtained from opera-exposed mice showed only minimal leukocyte infiltration. Furthermore, we observed that opera exposure in transplant recipients induced suppression of IL-2 and IFN-γ production and up-regulation of IL-4 and IL-10 in their splenocytes. IL-10 has anti-inflammatory and suppressive effects on most hematopoietic cells and is thus involved in the control of immune responses [27]. In the light of these findings, it appears possible that opera-induced regulatory cells may inhibit immune responses against allografts.

A second possible mechanism for the opera-induced hyporesponsiveness is the effects on brain function produced by the specific harmony and/or other features of the music itself. In our model, exposure to none of six single sound frequencies was associated with prolongation of allograft survival. Moreover, CBA recipients pre-treated with tympanic membrane perforation exposed to opera music rejected their graft acutely. Previous studies have indicated that frequency discrimination in humans is correlated with several cognitive skills, including facility with language [28], working memory [29], and ability to learn [30]. Moreover, frequency is represented explicitly and predominantly in individual neurons in the human auditory cortex [31]. However, little is known about possible mechanisms by which a single sound frequency might modulate alloimmune responses, and it has not been well determined that whether hearing loss induced by some immunosuppressants such as tacrolimus affects the graft survival [32]. In a previous study, exposure to music significantly enhanced levels of brain-derived neurotrophic factor and decreased levels of nerve growth factor in the hypothalamus of mice [33]. These findings suggest that music can influence brain function and development and that auditory stimulation from music, including specific harmony, may affect allograft survival.

A decrease in postoperative stress brought about by exposure to music is a third possible mechanism for the opera-induced hyporesponsiveness observed in our study. Stress has an important role in the development of symptoms and disease [34,35]. Zhuang *et al. *[36] found that endogenous stress caused by heterotopic heart transplantation contributed to postoperative cardiac injury and allograft vasculopathy in rats. In experiments in hypertensive rats, exposure to music composed by Mozart significantly decreased heart rate and had no effect on blood pressure, thereby producing a small decrease in cardiac output, whereas music composed by Ligeti significantly increased blood pressure but reduced heart rate. The effects of music could not be attributed to a stress reaction because stress caused by switching cages increased both heart rate and blood pressure in the animals [37].

A fourth possible mechanism for the opera-induced hyporesponsiveness in our model is that exposure to music increased, rather than decreased, stress. Numerous studies have found that stress can suppress the immune response and thereby be detrimental to health [38]. In a study in mice, Wistar and Hildemann [39] found that chronic avoidance-learning stress depressed the immune reaction responsible for rejection of skin homografts to a moderate but significant degree. In another murine model, Nùñez *et al. *[40] observed that a chronic auditory stressor induced a significant reduction in both natural killer cell activity and in vivo and in vitro generation of cytotoxic T lymphocytes. In a rat model, however, neutrophils and macrophages from animals exposed to noise for a short period secreted significantly less superoxide anion and IL-1 than cells from control rats, but lymphocyte function remained unchanged [41]. In our model, the presence of music-induced stress was unlikely because the mice exposed to opera did not lose hair or weight and the mean weight of their adrenal glands at sacrifice was not different from that of mice in the no-treatment group (data not shown).

## Conclusions

In summary, in a murine model, exposure to opera music had immuno-modulatory effects that resulted in prolonged survival of fully allogeneic grafts and generation of regulatory cells. Brain function with auditory stimulation may affect aspects of the peripheral immune response such as generation of regulatory CD4^+^CD25^+ ^cells and up-regulation of anti-inflammatory cytokines, resulting in prolonged allograft survival. Subsequent research on whether exposure to music is useful in suppressing the rejection reaction in organ transplantation, as well as on the type of music and the extent of music exposure that are most effective, must include studies in large animals.

## List of abbreviations

ALP: alkaline phosphatase; ELISA: enzyme-linked immunosorbent assay; Foxp3^+^: Forkhead box P3; IL: interleukin; IFN: interferon; mAb: monoclonal antibody; MACS: magnetically activated cell sorter; MLC: mixed leukocyte culture; MMC: mitomycin C; MST: median survival time.

## Competing interests

The authors declare that they have no competing interests.

## Authors' contributions

MN, QZ, and MU participated in research design, QZ, TH, HB and MU performed the experiments, MN XJ and MU participated in the writing of the manuscript, and XJ, AA and MU participated in data analysis. All authors read and approved the final manuscript.
